# High-altitude hypoxic cues and cerebral ischemic tolerance: an evidence-graded translational framework for stroke research

**DOI:** 10.3389/fnins.2026.1860879

**Published:** 2026-07-03

**Authors:** Xusheng Wu, Haijie Wang, Shuang Liang, Zongyu Cao, Jie Liu

**Affiliations:** 1 Clinical Medical College, Qinghai University, Xining, Qinghai, China; 2 Research Center for High Altitude Medicine, Qinghai University, Xining, Qinghai, China; 3 Department of Emergency Internal Medicine, Qinghai Provincial People’s Hospital, Xining, Qinghai, China

**Keywords:** blood–brain barrier, cerebral ischemic tolerance, high altitude, hypoxic preconditioning, neurovascular unit, spatial transcriptomics

## Abstract

High altitude exposes the brain to heterogeneous hypoxic, hemodynamic, rheological, inflammatory, and healthcare-access conditions. This heterogeneity makes altitude biologically informative for stroke research, but it does not justify treating natural altitude exposure as a single protective or harmful state. In this structured narrative review, we searched and organized the literature to ask which altitude-associated hypoxic cues resemble or reveal mechanisms compatible with cerebral ischemic tolerance, and what level of evidence supports that claim. We separate long-term adaptation, short-term acclimatization, chronic or excessive environmental hypoxia, and experimental hypoxic conditioning; define direct, supportive, and indirect evidence tiers; and integrate neurovascular-unit biology with multi-omics and stroke pathophysiology. Experimental hypoxic preconditioning remains the clearest direct evidence that a defined sublethal hypoxic stimulus can induce a time-limited tolerant state. In contrast, human high-altitude epidemiology, physiology, and genetics mainly constrain the clinical context and nominate candidate pathways rather than prove stroke-specific protection. We also emphasize that chronic hypoxia can be maladaptive through endothelial dysfunction, oxidative stress, erythrocytosis, thrombogenicity, blood–brain barrier impairment, and microvascular injury. Across neurovascular-unit cell types, a transparent evidence-weighting framework prioritizes endothelial biology because of its direct connection to BBB stability, effective reperfusion, hemorrhagic transformation risk, and no-reflow, while neurons, astrocytes, microglia, oligodendrocyte-lineage cells, and pericytes require different degrees of causal and human validation. We argue that the most productive path forward is not to label altitude as protective, but to use altitude-related biology to prioritize testable, stroke-facing hypotheses regarding BBB stability, microvascular patency, metabolic support, inflammatory thresholds, white-matter resilience, and biomarker-defined conditioning windows.

## 1 Introduction

### 1.1 Why high altitude is informative for translational stroke research

High altitude is useful to stroke research not because it offers a single risk narrative, but because it exposes the brain to competing biological and clinical forces. Reduced inspired oxygen pressure coexists with altered ventilation, erythropoietic change, rheological stress, endothelial activation, dehydration tendency, sleep disturbance, variable physical workload, and unequal access to acute stroke care (Duan et al., 2025; Zheng et al., 2023; Ortiz-Prado et al., 2022; Syed et al., 2022; Zhu et al., 2021; Wang et al., 2024; Ortiz-Prado et al., 2021; Tang et al., 2025). Some of these changes may favor compensation; others may increase vulnerability. The translational challenge is therefore not to ask whether altitude is globally protective, but whether specific altitude-associated hypoxic cues engage programs compatible with cerebral ischemic tolerance.

That distinction matters for a stroke journal. A natural exposure can be mechanistically informative even when it does not behave like a therapeutic intervention. Human high-altitude data may reveal where compensation and injury coexist, identify which cell systems carry the strongest adaptive signal, and help define which patient strata are unlikely to benefit from conditioning-like strategies. These are stroke-facing questions because they bear directly on reperfusion success, blood–brain barrier (BBB) integrity, tissue salvage, and the design of biomarker-guided interventions (Duan et al., 2025; Zheng et al., 2023; Ortiz-Prado et al., 2022; Syed et al., 2022; Zhu et al., 2021; Wang et al., 2024; Ortiz-Prado et al., 2021; Tang et al., 2025).

### 1.2 Conceptual boundaries: adaptation, acclimatization, chronic exposure, and conditioning are related but not interchangeable

A first step is to separate biological claims that are often blurred together. High-altitude adaptation refers to population-level genetic and phenotypic traits shaped by long-term selection and lifelong exposure, with EPAS1 and EGLN1 among the best characterized examples (Li et al., 2023; Simonson et al., 2010; Yi et al., 2010). Acclimatization refers to reversible physiological adjustment after ascent in lowlanders, including ventilatory, circulatory, and hemoglobin-related responses (Hoiland et al., 2018; Baillieul et al., 2017). Chronic environmental hypoxia refers to prolonged ambient exposure with heterogeneous altitude, duration, temperature, dehydration, sleep, workload, access-to-care, and comorbidity contexts; it may include both compensatory and injury-promoting biology. Experimental hypoxic preconditioning (HPC) refers to a defined sublethal stimulus administered to induce tolerance to a later hypoxic or ischemic insult. Intermittent hypoxia (IH), intermittent hypoxia-hyperoxia exposure (IHHE), ischemic preconditioning (IPC), and remote ischemic conditioning (RIC/RIPC) are related intervention paradigms, but they are not equivalent to living at altitude (Baillieul et al., 2017; Yuan et al., 2022; Ran et al., 2005; Zhu et al., 2025; Hao et al., 2020; Keevil et al., 2024; Li et al., 2024; Chen et al., 2022; Wang et al., 2024).

This boundary prevents a common translational error. Evidence that high-altitude residents carry adaptive traits does not prove that they are in a stroke-resistant state at the moment ischemia occurs. Evidence that lowlanders acclimatize after ascent does not show that acclimatization reproduces a preconditioning program. Evidence that HPC protects rodent brain tissue does not establish that natural altitude exposure has induced the same state in humans. The relevant question is therefore conditional and mechanistic: under which exposure pattern, in which tissue niche, in which time window, and with what endpoint does a hypoxia-related program behave as cerebral ischemic tolerance? The terminology used throughout this review is summarized in Box 1.

### 1.3 What multi-omics adds to a stroke-facing framework

Multi-omics is valuable here because ischemic tolerance is unlikely to be a uniform whole-brain state. It is more plausibly assembled from cell-state changes in defined anatomical niches and within specific time windows. Single-cell RNA sequencing (scRNA-seq) is especially useful in experimental systems that need fine cellular resolution. Single-nucleus RNA sequencing (snRNA-seq) is particularly powerful in ischemic, frozen, postmortem, or glia-rich tissue. Spatial transcriptomics contributes anatomical localization and helps determine whether candidate sender and receiver populations occupy the same stroke-relevant territory (Haque et al., 2017; Lee et al., 2022; Nguyen et al., 2018; Zhang Y. et al., 2024; Liu et al., 2025; Deng et al., 2025; Zhang L. et al., 2024; Lv et al., 2025; Zhang et al., 2026; Han et al., 2024; Garcia-Bonilla et al., 2024; Bormann et al., 2024; Zhao et al., 2026).

These approaches have shifted the field in important ways. They have made neuronal vulnerability appear subtype- and region-selective rather than generic, replaced binary glial labels with state-rich architectures, and highlighted white matter, peri-infarct cortex, penumbra, and hippocampal niches as biologically distinct territories rather than passive background (Zhang Y. et al., 2024; Liu et al., 2025; Deng et al., 2025; Zhang L. et al., 2024; Lv et al., 2025; Zhang et al., 2026; Han et al., 2024; Garcia-Bonilla et al., 2024; Bormann et al., 2024; Zhao et al., 2026). At the same time, omics should not be mistaken for mechanism. Differential expression, pseudotime, and ligand-receptor prediction generate prioritized hypotheses, but they do not prove causal protection unless they are followed by spatial validation, perturbation, and rescue (Lee et al., 2022; Nguyen et al., 2018; Zhang Y. et al., 2024; Liu et al., 2025; Deng et al., 2025; Zhang L. et al., 2024; van den Brink et al., 2017; Luecken and Theis, 2019; Tran et al., 2020; Armingol et al., 2021).

### 1.4 Review scope, literature search, and evidence-grading methodology

This article was designed as a structured narrative and evidence-graded translational review rather than a systematic review or meta-analysis. The aim was not to quantitatively estimate the effect of altitude exposure or conditioning interventions on stroke outcomes, but to organize the available literature into a framework that distinguishes established mechanisms, plausible hypotheses, indirect context-setting evidence, and remaining translational gaps.

We searched PubMed/MEDLINE, Web of Science, Embase, Scopus, and Google Scholar from database inception to the final manuscript update in June 2026. Search terms included combinations of “high altitude,” “hypoxia,” “chronic hypoxia,” “hypobaric hypoxia,” “hypoxic preconditioning,” “intermittent hypoxia,” “intermittent hypoxia-hyperoxia,” “ischemic preconditioning,” “remote ischemic conditioning,” “cerebral ischemia,” “ischemic stroke,” “neurovascular unit,” “blood–brain barrier,” “endothelial cell,” “astrocyte,” “microglia,” “oligodendrocyte,” “pericyte,” “single-cell RNA sequencing,” “single-nucleus RNA sequencing,” and “spatial transcriptomics.” Reference lists of key reviews and mechanistic studies were also screened manually.

Studies were prioritized if they addressed one or more of the following domains: defined hypoxic or ischemic conditioning paradigms with cerebral ischemia-relevant endpoints; mechanistic studies of hypoxia-responsive pathways in stroke-relevant cells or tissues; human altitude epidemiology, physiology, or genetics relevant to cerebrovascular risk or adaptation; and omics studies that localized candidate cell states or pathways in stroke- or hypoxia-relevant tissue compartments. Studies were excluded if they lacked relevance to the central nervous system or stroke-facing vascular biology, did not clearly define hypoxic or ischemic exposure, or could not be linked to ischemic tolerance, neurovascular-unit function, or translational stroke endpoints.

**Figure 1:**
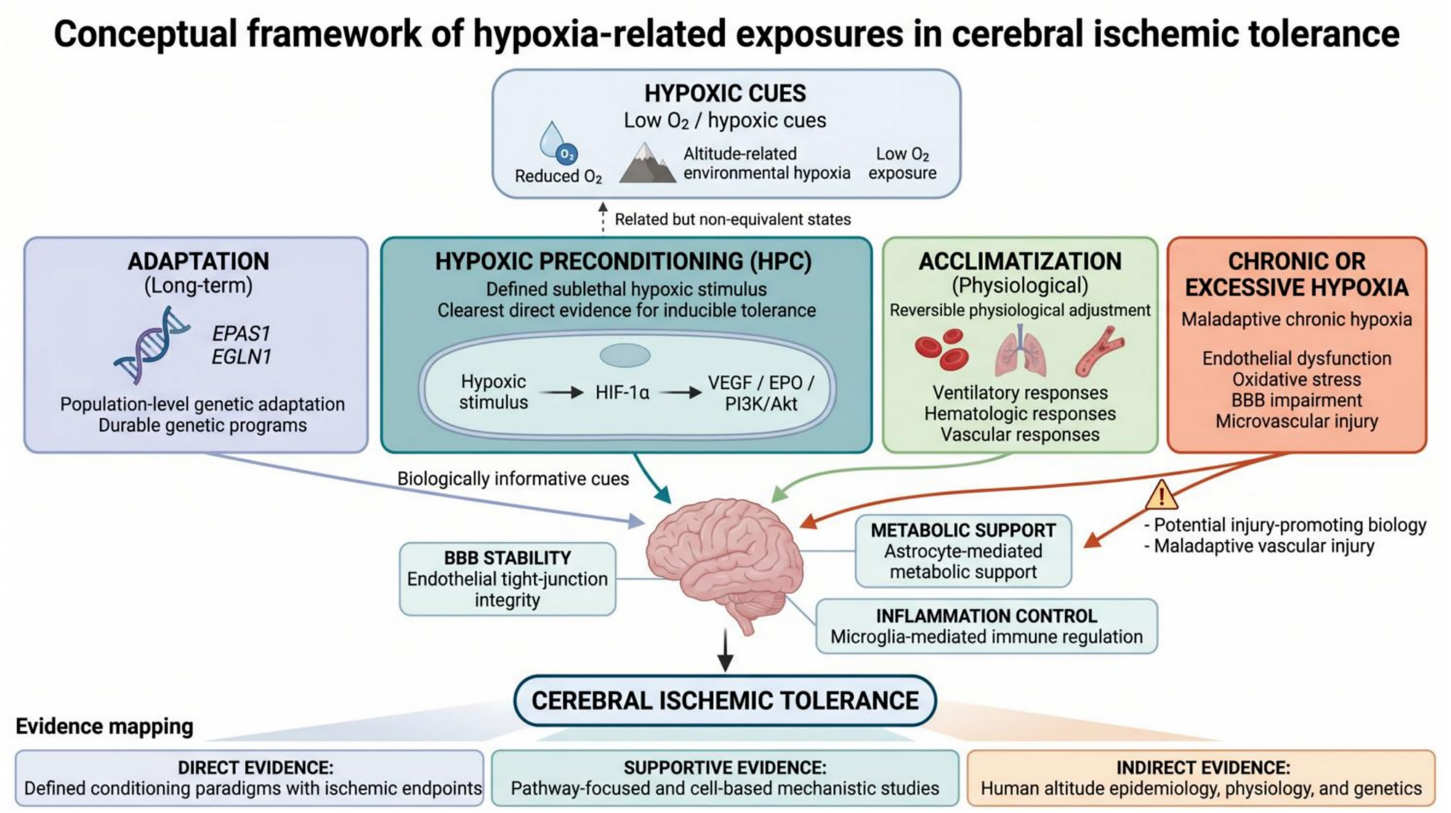
Revised conceptual framework distinguishing natural altitude-related exposure from controlled experimental conditioning. The figure separates long-term adaptation, acclimatization, chronic or excessive hypoxia, and defined conditioning paradigms, while emphasizing that shared hypoxia-responsive cues do not imply biological equivalence. The lower panel indicates how exposure classes map onto direct, supportive, and indirect evidence tiers.

Evidence assignment followed operational criteria rather than quantitative meta-analytic weighting. Direct evidence required a defined conditioning paradigm, an ischemic or stroke-relevant endpoint, and, where available, tissue or cell-type localization. Supportive evidence included pathway-focused, cell-based, perturbation, rescue, or intervention studies that strengthened causal plausibility but did not reproduce the full stroke or altitude context. Indirect evidence included human altitude epidemiology, physiology, genetics, and comparative adaptation biology that defined exposure heterogeneity, clinical constraints, and candidate pathways but could not establish stroke-specific preconditioning on its own. Because included exposures, species, models, endpoints, and clinical contexts were heterogeneous, no pooled effect estimate was attempted (Figure 1, Table 1).

Box 1 Terminology used to avoid conflating exposure and intervention paradigms.

**Table:** 

Term	Operational meaning in this review
High-altitude adaptation	Population-level genetic and phenotypic traits shaped by long-term selection or lifelong exposure; useful for candidate pathway discovery but not proof of acute stroke protection.
Acclimatization	Reversible physiological adjustment after ascent in lowlanders, including ventilatory, circulatory, hemoglobin-related and vascular responses.
Chronic environmental hypoxia	Prolonged natural exposure with heterogeneous dose, duration and context; may produce compensatory and maladaptive vascular or hematologic effects.
HPC	Defined sublethal hypoxic stimulus before a later hypoxic or ischemic insult; strongest direct evidence for inducible ischemic tolerance when stroke endpoints are measured.
IH and IHHE	Repeated cycles of hypoxia alone or hypoxia alternating with hyperoxia; intervention paradigms requiring explicit dose, interval, duration and safety definition.
IPC	A brief ischemic stimulus applied before a later ischemic insult; mechanistically related to HPC but not identical.
RIC/RIPC	Remote limb or tissue ischemia cycles intended to induce systemic protection; promising but workflow-, timing-, and patient-selection dependent.

**Table 1:** Operational evidence hierarchy for hypoxia-related cerebral ischemic tolerance.

Evidence tier	Operational definition	What it can support	Main limitations
Direct	Defined HPC or related conditioning paradigm with cerebral ischemia-relevant endpoints, preferably with stroke-relevant tissue, cell-type or spatial localization (Zhu et al., 2025; Haque et al., 2017; Zhang Y. et al., 2024; Liu et al., 2025; Deng et al., 2025; Han et al., 2024; Garcia-Bonilla et al., 2024; Bormann et al., 2024; Zhao et al., 2026; Darlington et al., 2021; Zhao et al., 2020).	That a defined hypoxic or conditioning stimulus can induce a time-limited tolerant state under controlled conditions; which cell types and tissue niches are plausible mediators.	Usually preclinical; often young and otherwise healthy models; localization alone does not prove mechanism; clinical workflow is not captured.
Supportive	Pathway-focused HPC/IPC/IH/IHHE studies, cell-based mechanistic work, perturbation and rescue experiments, and intervention studies that strengthen causal plausibility (Baillieul et al., 2017; Yuan et al., 2022; Ran et al., 2005; Zhu et al., 2025; Hao et al., 2020; Keevil et al., 2024; Li et al., 2024; Chen et al., 2022; Wang et al., 2024; Zhao et al., 2020; Mitroshina et al., 2021; Huang et al., 2020; Zhan et al., 2021; Lu et al., 2024; Zheng et al., 2024; Ruscher et al., 2002; Dong et al., 2022; Qiu et al., 2024; Guo et al., 2019; Wu et al., 2021; Fadoul et al., 2024; Coimbra-Costa et al., 2021; Hirayama et al., 2024; An and Xue, 2009; Wacker et al., 2012; Zhang P. et al., 2024).	Biological plausibility, candidate pathways, and communication loops that warrant testing in stroke models or human tissue.	May not reproduce altitude exposure, comorbidity, stroke subtype, reperfusion variability, or human tissue architecture.
Indirect	Human altitude epidemiology, physiology, genetics, and comparative adaptation biology (Duan et al., 2025; Zheng et al., 2023; Ortiz-Prado et al., 2022; Syed et al., 2022; Zhu et al., 2021; Wang et al., 2024; Ortiz-Prado et al., 2021; Tang et al., 2025; Li et al., 2023; Simonson et al., 2010; Yi et al., 2010; Hoiland et al., 2018; Stacey et al., 2023; Berek et al., 2025; Zhao et al., 2021).	Exposure heterogeneity, clinical constraints, candidate pathways, and patient strata in which compensation or injury may dominate.	Cannot establish a stroke-specific preconditioned human phenotype or prove that natural altitude exposure is protective.

## 2 What the present evidence does and does not establish

### 2.1 Experimental hypoxic preconditioning remains the clearest direct evidence

If the narrow question is whether hypoxic stimulation can induce cerebral ischemic tolerance, the clearest answer still comes from experimental HPC. Across preclinical models, a sublethal hypoxic stimulus can improve subsequent outcome after focal or global ischemia within a defined temporal window. This is important because it establishes tolerance as an inducible and time-dependent state rather than a permanent property. It also implies that exposure duration cannot be treated as a simple more-is-better continuum: a short conditioning paradigm and a prolonged hypoxic exposure may represent biologically different states, including mixed adaptive-injury states at longer durations (Darlington et al., 2021; Zhao et al., 2020).

Mechanistically, HPC is best understood as a distributed response rather than a single master switch. HIF-centered transcription interacts with VEGF-, erythropoietin (EPO)-, and DDAH-1-related signaling; PI3K/Akt survival pathways; and programs affecting neurogenesis, apoptosis, necroptosis, and inflammatory tone (Zhao et al., 2020; Mitroshina et al., 2021; Huang et al., 2020; Zhan et al., 2021; Lu et al., 2024; Zheng et al., 2024; Ruscher et al., 2002; Dong et al., 2022; Qiu et al., 2024). This body of work supports a strong conclusion directly relevant to stroke translation: hypoxia can precondition brain tissue under defined conditions. It does not prove that naturally occurring altitude exposure has already created the same state in humans.

### 2.2 Human high-altitude studies constrain tolerance claims rather than prove them

Human altitude studies are highly informative, but their strongest contribution is to define the exposure landscape in which tolerance claims must be constrained. Systematic reviews and regional analyses suggest that stroke occurrence and outcome at moderate or moderately high altitude are not uniformly worse and may in some settings be associated with lower stroke mortality (Zheng et al., 2023; Ortiz-Prado et al., 2022; Syed et al., 2022; Ortiz-Prado et al., 2021). At higher elevations, however, erythrocytosis, hyperviscosity, endothelial stress, coagulation shifts, dehydration, and delayed access to specialized care may outweigh compensation (Duan et al., 2025; Syed et al., 2022; Zhu et al., 2021; Wang et al., 2024; Ortiz-Prado et al., 2021; Tang et al., 2025). A nonlinear altitude-exposure-risk relationship is therefore more defensible than either a monotonic protective narrative or a monotonic harmful one.

These studies matter because they identify where a conditioning-compatible signal could plausibly coexist with major translational barriers such as ineffective reperfusion, delayed presentation, and microvascular dysfunction. They also show that stroke subtype matters. Intracerebral hemorrhage at altitude may have a different clinical profile from ischemic stroke, and high-altitude-associated ischemic stroke in younger adults may reflect a partially distinct pathophysiological mix (Syed et al., 2022; Zhu et al., 2021; Wang et al., 2024; Ortiz-Prado et al., 2021; Tang et al., 2025). These studies are therefore predominantly indirect: they are essential for boundary setting and patient stratification, but they do not establish a preconditioned human phenotype.

### 2.3 Physiological and genetic studies nominate pathways rather than stroke-specific protection

Physiological and genetic studies move closer to mechanism but still stop short of demonstrating stroke-specific tolerance. Acclimatization can buffer hypoxic stress through altered ventilation, cerebrovascular regulation, and oxygen transport (Hoiland et al., 2018; Baillieul et al., 2017). Long-term high-altitude populations exhibit more durable phenotypes, with distinct hemoglobin set points, ventilatory responses, and adaptive architectures involving EPAS1, EGLN1, and coordinated systems-level physiology (Li et al., 2023; Simonson et al., 2010; Yi et al., 2010). These findings make it clear that humans can deploy robust hypoxia-responsive biology. What they do not show is that these responses are configured as tissue-protective programs at the time of cerebral ischemia.

Several observations deserve priority in a translational framework. Lifelong high-altitude exposure has been associated with improved redox homeostasis and structural-functional adaptation of the neurovascular unit (Stacey et al., 2023). Simulated high-altitude studies linking higher EPO with lower neurofilament light suggest that neuroprotective signaling may be active *in vivo* (Berek et al., 2025). Comparative work has also implicated Akt-linked neuroprotective responses in high-altitude adaptation biology (Zhao et al., 2021). These data are best interpreted as supportive-to-indirect evidence: they strengthen biological plausibility, point toward candidate pathways, and help decide which mechanisms are worth validating in stroke models and human tissue.

### 2.4 Maladaptive chronic hypoxia and boundary conditions

A critical boundary of the present framework is that hypoxia-responsive biology should not be equated with protection. Controlled conditioning paradigms and chronic environmental hypoxia differ fundamentally in oxygen dose, duration, intermittency, hematologic response, vascular adaptation, inflammatory tone, and clinical context. While defined hypoxic preconditioning can induce tolerance under experimental conditions, chronic or excessive hypoxia may promote endothelial dysfunction, oxidative and nitrosative stress, erythrocytosis, hyperviscosity, thrombogenicity, leukocyte-endothelial activation, BBB disruption, microvascular injury, and impaired tissue reperfusion (Duan et al., 2025; Syed et al., 2022; Zhu et al., 2021; Wang et al., 2024; Ortiz-Prado et al., 2021; Tang et al., 2025; Li et al., 2025; Iadecola, 2017; Sweeney et al., 2019; Loan et al., 2024; Roth et al., 2025; Sun et al., 2024).

These maladaptive effects are especially relevant in patients with hypertension, diabetes, atherosclerosis, small-vessel disease, dehydration, obstructive sleep-disordered breathing, polycythemia, or delayed access to acute stroke care. The same HIF-, VEGF-, EPO-, PI3K/Akt-, Nrf2-, or inflammatory pathway may therefore be adaptive during a defined preconditioning window but neutral or harmful when excessive, prolonged, delayed, or activated during established ischemic injury. Altitude-associated biology should accordingly be interpreted as a source of mechanistic cues and translational constraints, not as evidence that natural altitude exposure is intrinsically protective.

## 3 Neurovascular-unit programs most relevant to translation

### 3.1 Evidence weighting across neurovascular-unit cell types

For translational stroke research, the neurovascular unit is the most useful integrative frame because tissue survival depends on more than neuronal viability alone. Penumbral salvage also depends on endothelial integrity, leukocyte-endothelial interaction, astrocytic metabolic support, microglial inflammatory thresholds, white-matter resilience, and the persistence of capillary perfusion (Zhu et al., 2025; Lv et al., 2025; Zhang et al., 2026; Han et al., 2024; Garcia-Bonilla et al., 2024; Bormann et al., 2024; Zhao et al., 2026; Li et al., 2025; Iadecola, 2017; Sweeney et al., 2019). Multi-omics strengthens this view by assigning vulnerability and compensation to defined cell states and locations rather than broad anatomical compartments.

To reduce interpretive ambiguity, we reassessed cell-type maturity using five criteria: defined conditioning evidence, cerebral ischemia or stroke-relevant endpoints, perturbation or rescue evidence, spatial or cell-type localization, and human relevance or clinically measurable endpoints. Each criterion was scored ordinally from 0 to 2, where 0 indicated absent or indirect support, 1 indicated supportive but incomplete evidence, and 2 indicated strong or convergent evidence. The total score is not a meta-analytic effect size; it is a transparent prioritization tool that makes explicit why some mechanisms are more ready for near-term translational testing than others (Table 2).

**Table 2:** Evidence-weighting framework across neurovascular-unit cell types.

Cell type/axis	Evidence score	Main evidence basis	Translational interpretation
Endothelial cells	9/10	Defined preconditioning evidence for junctional protection and adhesion-molecule reduction; strong stroke relevance through BBB integrity, reperfusion, hemorrhagic transformation risk and no-reflow (Li et al., 2025; Iadecola, 2017; Sweeney et al., 2019; An and Xue, 2009; Wacker et al., 2012; Loan et al., 2024; Roth et al., 2025; Sun et al., 2024).	Highest near-term translational priority because endpoints are clinically measurable and directly related to reperfusion-era stroke care.
Neurons	8/10	Direct readout of ischemic tolerance; evidence for PI3K/Akt, HIF-1α, VEGF, EPO, apoptosis, necroptosis, neurogenesis and plasticity (Huang et al., 2020; Zhan et al., 2021; Lu et al., 2024; Zheng et al., 2024; Ruscher et al., 2002; Dong et al., 2022).	Necessary readout of protection, but insufficient alone unless integrated with vascular and glial mechanisms.
Astrocytes	8/10	Strong convergence around lactate support, glutamate handling, mitochondrial stabilization, antioxidant buffering, Nrf2, HIF-2 and EPO programs (Ma et al., 2022; Escartin et al., 2021; Guo et al., 2019; Wu et al., 2021; Fadoul et al., 2024; Coimbra-Costa et al., 2021; Hirayama et al., 2024).	High priority for penumbral metabolic support and region-specific injury-threshold modulation.
Microglia/macrophages	7/10	Support for inflammatory threshold regulation, NF-κB/NLRP3, IL-1R1-p-MLKL, A20-RIP3 and state-specific post-stroke responses (Zhan et al., 2021; Lu et al., 2024; Qiu et al., 2024; Ransohoff, 2016; Masuda et al., 2020; Ma et al., 2023; McDonough et al., 2020).	Moderate-to-high maturity, but translation requires time-window and state-specific targeting rather than broad anti-inflammatory suppression.
Oligodendrocyte-lineage cells	5/10	Relevant to white-matter integrity and network function; evidence from hypoxic–ischemic and omics studies is encouraging but less mature in adult stroke (Lv et al., 2025; Zhao et al., 2026; Xu et al., 2019; Suryana and Jones, 2014; Kuwata et al., 2025; Roth and Núñez, 2016).	Promising for disability and cognitive outcomes; requires adult stroke validation and causal testing.
Pericytes	5/10	Highly relevant to BBB integrity, capillary tone and no-reflow; stroke heterogeneity evidence is increasing, but direct hypoxic-conditioning evidence is sparse (Li et al., 2025; Loan et al., 2024; Roth et al., 2025; Sun et al., 2024).	High-value frontier, especially when paired with endothelial and capillary-flow endpoints.

### 3.2 Neurons remain the most direct readout of tolerance, but not a uniform target

Neurons remain the clearest cellular readout of whether ischemic tolerance has been achieved. Omics data from stroke and high-altitude-related injury indicate that neuronal vulnerability is not uniform: glutamatergic populations, hippocampal and dentate-gyrus neurons, and energetically demanding excitatory circuits may be particularly sensitive to mitochondrial stress, calcium dysregulation, and synaptic collapse (Lv et al., 2025; Zhang et al., 2026; Han et al., 2024; Garcia-Bonilla et al., 2024). This refines the translational question from whether neurons matter to which neuronal populations have their injury threshold shifted.

Functional evidence remains strong. HPC and IPC engage PI3K/Akt-associated survival signaling, reduce apoptosis and necroptosis, and improve neurogenesis and post-ischemic plasticity through HIF-1α-, VEGF-, and EPO-related pathways (Huang et al., 2020; Zhan et al., 2021; Lu et al., 2024; Zheng et al., 2024; Ruscher et al., 2002; Dong et al., 2022). For stroke translation, neuronal protection is necessary but not sufficient: it is the most established sign that a tolerant state exists, but it must be integrated with vascular and glial protection if it is to remain meaningful under reperfusion conditions.

### 3.3 Astrocytes bridge metabolic support, glutamate control, and local inflammatory amplification

Astrocytes occupy a central position in any stroke-facing tolerance model because they regulate glutamate uptake, lactate shuttling, antioxidant buffering, ionic homeostasis, and mitochondrial support. Modern omics has replaced the old A1/A2 shorthand with a richer spectrum of reactive astrocyte states that vary across region, disease stage, and injury severity (Ma et al., 2022; Escartin et al., 2021). This matters because a translationally relevant astrocyte state is not simply ‘activated’; it is either stabilizing or destabilizing the local injury threshold.

This is one of the strongest areas of convergence between functional studies and omics. Hypoxic conditioning enhances antioxidant capacity, stabilizes astrocytic mitochondrial function, and engages PGC-1α/HIF-, Nrf2-, HIF-2-, and EPO-related programs (Guo et al., 2019; Wu et al., 2021; Fadoul et al., 2024; Coimbra-Costa et al., 2021). Preconditioning-induced facilitation of astrocytic lactate release provides a concrete mechanism by which metabolic support may delay neuronal energetic collapse (Hirayama et al., 2024). Spatial data also sharpen this picture: IL-1α-enriched reactive astrocytes associated with reduced SLC1A2 in the dentate gyrus link inflammatory amplification, impaired glutamate handling, and a defined vulnerable niche (Zhang et al., 2026). For translational stroke research, astrocytes therefore represent both a mechanistic bridge and a tractable prioritization problem: which states should be preserved, in which territories, and at which time points?

### 3.4 Microglia regulate the inflammatory threshold of tissue failure

Microglia are important not merely because they react to ischemia, but because they help determine whether tissue stress becomes containable or self-amplifying. Single-cell studies consistently show multidimensional microglial states involving inflammatory signaling, antigen presentation, phagocytosis, interferon response, and proliferation rather than a stable M1/M2 dichotomy (Garcia-Bonilla et al., 2024; Bormann et al., 2024; Ransohoff, 2016; Masuda et al., 2020; Ma et al., 2023). In a translational context, this shifts the goal from classifying activation to identifying threshold-setting states.

Supportive conditioning data suggest that microglia participate in building tolerance before injury occurs. Ischemic preconditioning can trigger microglial proliferation and cell-cycle activation in cortex, while HPC and IPC suppress inflammatory escalation through NF-κB/NLRP3, IL-1R1-p-MLKL, and A20-RIP3-linked pathways (Zhan et al., 2021; Lu et al., 2024; Qiu et al., 2024; McDonough et al., 2020). The translational implication is twofold. First, inflammation control should be framed as timing- and state-dependent rather than as blanket suppression. Second, microglial programs are most persuasive when tied to tissue outcome and local communication circuits rather than to catalogues of reactive phenotypes.

### 3.5 Endothelial cells should be treated as a primary translational axis

Among non-neuronal mechanisms, the endothelial compartment deserves special emphasis because it sits at the interface between conditioning biology and clinically meaningful tissue salvage. Endothelial cells regulate BBB integrity, leukocyte adhesion, capillary patency, and the quality of reperfusion. In a reperfusion era, a neuroprotective signal that fails to stabilize the microvasculature is unlikely to convert into effective tissue rescue (Han et al., 2024; Li et al., 2025; Iadecola, 2017; Sweeney et al., 2019; An and Xue, 2009; Wacker et al., 2012; Loan et al., 2024; Roth et al., 2025; Sun et al., 2024).

Functional evidence is compelling. Preconditioning preserves junctional proteins, reduces ICAM-1 and VCAM-1 expression, and attenuates reperfusion-associated barrier leakage (An and Xue, 2009; Wacker et al., 2012). These changes map directly onto major translational problems in stroke, including BBB breakdown, leukocyte recruitment, capillary obstruction, no-reflow, and incomplete tissue reperfusion despite macrovascular recanalization (Li et al., 2025; Iadecola, 2017; Sweeney et al., 2019; Loan et al., 2024; Roth et al., 2025; Sun et al., 2024). For this reason, endothelial biology receives the strongest near-term translational priority in the revised evidence-weighting matrix. This priority does not mean that endothelial cells are the only important target; it means that the endothelial axis provides the clearest connection between hypoxia-related mechanisms and clinically measurable reperfusion-era endpoints.

### 3.6 Oligodendrocyte-lineage cells extend tolerance biology into white matter

Oligodendrocyte precursor cells (OPCs) and immature oligodendrocytes add a white-matter dimension that is increasingly important for functional outcome. These cells are vulnerable to oxidative stress, excitotoxicity, and iron imbalance, and their fate influences myelin preservation, network conduction, and later cognitive recovery (Xu et al., 2019; Suryana and Jones, 2014; Kuwata et al., 2025; Roth and Núñez, 2016). This is relevant because stroke disability often reflects injury to distributed networks rather than gray matter loss alone.

Recent data are encouraging but still comparatively early. Severe hypoxia can induce pro-angiogenic OPC states, and HPC can support OPC maturation and white-matter integrity in hypoxic–ischemic models (Xu et al., 2019; Suryana and Jones, 2014; Kuwata et al., 2025). Omics also shows substantial oligodendrocyte-lineage remodeling in post-stroke human tissue and in high-altitude-related injury (Lv et al., 2025; Zhao et al., 2026). These data justify keeping oligodendrocyte biology within the tolerance framework, but they still support a more exploratory status than the endothelial, astrocytic, microglial, or neuronal axes in the specific context of hypoxia-induced adult cerebral ischemic tolerance.

### 3.7 Pericytes are a strategic frontier for no-reflow biology

Pericytes are well placed to influence capillary tone, basement membrane integrity, BBB stability, and microvascular persistence. Recent stroke studies show marked pericyte heterogeneity after ischemia and suggest that pericyte responses may precede endothelial death and barrier failure while also contributing to no-reflow (Li et al., 2025; Loan et al., 2024; Roth et al., 2025; Sun et al., 2024). This makes them highly relevant to the translational endpoint that most strongly links microvascular biology to patient outcome: effective, not merely angiographic, reperfusion.

**Figure 2:**
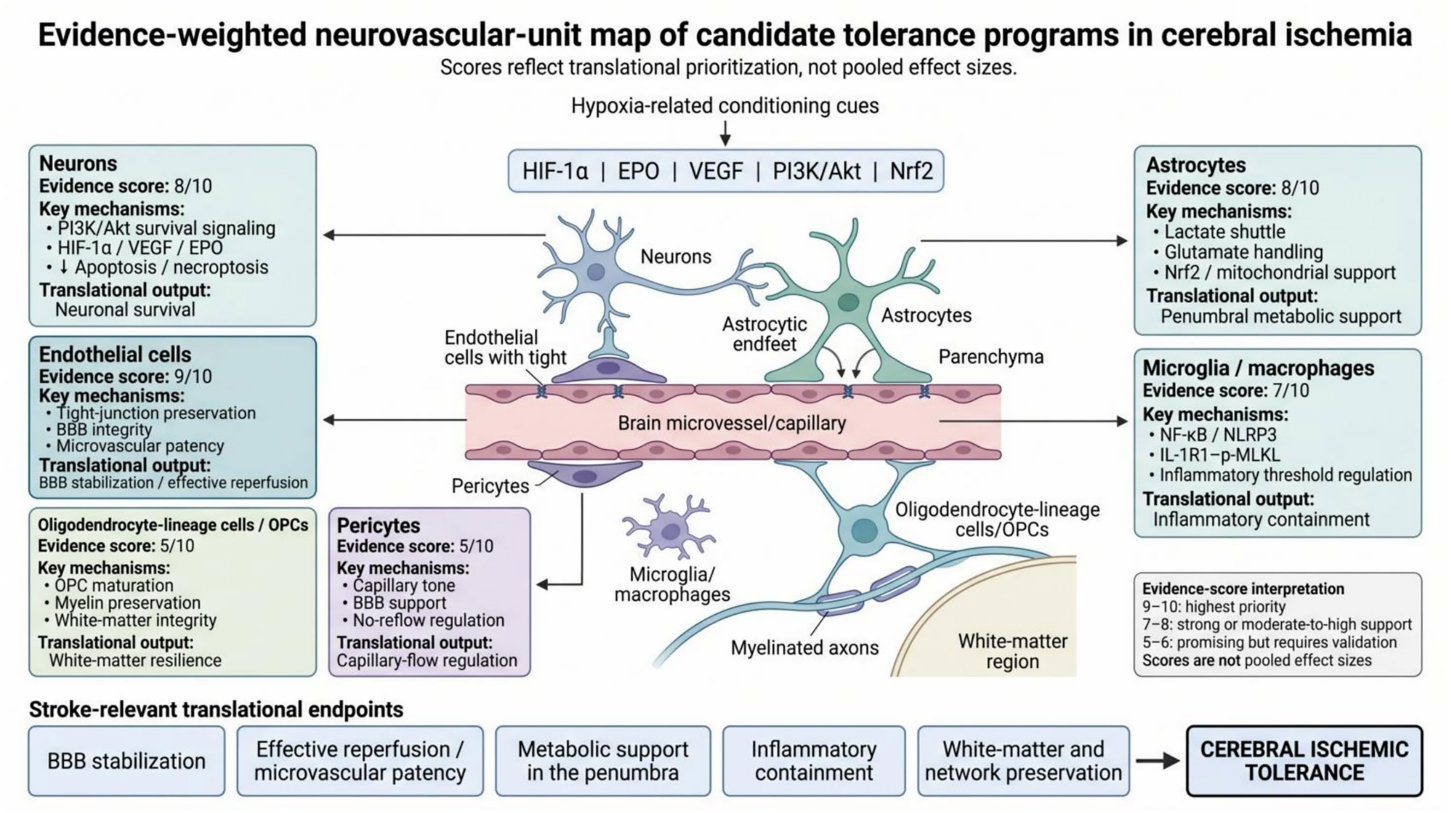
Evidence-weighted neurovascular-unit map of candidate tolerance programs in cerebral ischemia. The figure integrates cell-type evidence scores, candidate mechanisms, and translational endpoints. Scores are ordinal prioritization scores based on defined conditioning evidence, stroke endpoints, perturbation/rescue evidence, spatial or cell-type localization, and human or clinically measurable relevance; they are not pooled effect sizes.

Yet the evidence should still be graded carefully. Pericytes are clearly important to stroke pathophysiology, but direct evidence that hypoxic conditioning induces a protective pericyte state remains sparse. The most defensible current view is that pericytes represent a high-value frontier for tolerance research rather than an already mature effector layer. They are especially attractive when paired with endothelial readouts and capillary-flow endpoints (Figure 2).

## 4 A stroke-facing framework for interpretation and validation

### 4.1 Timing: the preconditioning window and the stroke timeline are different axes

A major source of translational confusion is the assumption that conditioning time and disease time are the same axis. Classical HPC is delivered before injury, with protection emerging and then fading over days (Darlington et al., 2021; Zhao et al., 2020). Clinical stroke unfolds through acute energy failure and excitotoxicity, followed by BBB disruption, inflammatory recruitment, microvascular dysfunction, and later glial, vascular, and white-matter remodeling. The same pathway may therefore be adaptive in a conditioning window yet maladaptive if excessive, delayed, or prolonged during disease evolution (Hirayama et al., 2024; Roth and Núñez, 2016; Ugale et al., 2025; Madai et al., 2024; Lanigan and O'Connor, 2019; Amin et al., 2021).

This temporal distinction should guide both mechanism and trial design. Statements that a pathway is ‘protective’ are incomplete unless they specify when protection is expected, in relation to stroke onset, thrombolysis, thrombectomy, reperfusion, and recovery phase. Conditioning-based strategies are most likely to fail when this temporal mismatch is ignored.

### 4.2 Region: tolerance should be localized to tissue niches, not the whole brain

Region is equally important. The ischemic core is characterized by irreversible energy collapse, whereas the penumbra retains conditional reversibility and is therefore the most plausible compartment in which tolerance biology can alter tissue fate (Liu et al., 2025; Deng et al., 2025; Han et al., 2024). White matter, peri-infarct cortex, and hippocampal territories add distinct priorities related to network dysfunction and delayed cognitive sequelae (Deng et al., 2025; Lv et al., 2025; Zhang et al., 2026; Zhao et al., 2026).

Spatially resolved studies make these distinctions operational. They localize candidate vulnerability and compensation programs to dentate-gyrus niches, peri-infarct cortex, penumbra, and white matter (Liu et al., 2025; Deng et al., 2025; Lv et al., 2025; Zhang et al., 2026; Han et al., 2024; Garcia-Bonilla et al., 2024; Bormann et al., 2024; Zhao et al., 2026). Translationally, this means that the relevant unit of analysis is not ‘the hypoxic brain’ but cell state within a specific stroke-relevant location. That is where future protective hypotheses should be framed and tested.

### 4.3 Cell–cell communication should be judged by an evidence ladder

Hypoxic conditioning is unlikely to work by independently optimizing every cell type. A more realistic model is that it reshapes communication among neurons, astrocytes, microglia, endothelial cells, pericytes, and circulating signals. Candidate examples include EPO- and VEGF-related signaling, astrocyte-derived lactate support, endothelial exosomal miR-126, and humoral pathways implicated in remote ischemic conditioning (Hao et al., 2020; Keevil et al., 2024; Ruscher et al., 2002; Dong et al., 2022; Hirayama et al., 2024; Zhang P. et al., 2024).

For translational purposes, communication claims should be ranked rather than treated as equivalent. A practical ladder is co-expression < spatial adjacency < perturbation < mechanistic rescue. Co-expression establishes possibility. Spatial adjacency adds anatomical plausibility. Perturbation indicates dependence. Rescue closes the loop and most strongly supports mechanism. This ranking is especially valuable in omics-driven literature, where communication is easy to predict but harder to prove (van den Brink et al., 2017; Luecken and Theis, 2019; Tran et al., 2020; Armingol et al., 2021).

### 4.4 Methodological caveats in omics-based inference

Omics technologies are powerful tools for localizing candidate cell states and generating mechanistic hypotheses, but they have important limitations. scRNA-seq may be affected by dissociation-induced transcriptional artifacts, selective loss of vulnerable cells, ambient RNA contamination, doublets, and ischemia-associated stress signatures introduced or amplified during tissue processing (Haque et al., 2017; Nguyen et al., 2018; van den Brink et al., 2017; Luecken and Theis, 2019; Tran et al., 2020). snRNA-seq is well suited for frozen, postmortem, or glia-rich tissues, but it may incompletely capture cytoplasmic transcripts and may not fully correspond to whole-cell transcriptional states. Spatial transcriptomics adds anatomical context, but its resolution, sensitivity, and cell-type deconvolution remain platform-dependent (Lee et al., 2022; Zhang Y. et al., 2024; Zhang L. et al., 2024).

Interpretation is further complicated by batch effects, cross-platform differences, species-specific transcriptional programs, model-specific ischemic responses, postmortem interval effects, and incomplete correspondence between transcriptomic states and protein abundance, metabolism, electrophysiology, or functional phenotype (Haque et al., 2017; Lee et al., 2022; Nguyen et al., 2018; Zhang Y. et al., 2024; Liu et al., 2025; Deng et al., 2025; Zhang L. et al., 2024; van den Brink et al., 2017; Luecken and Theis, 2019; Tran et al., 2020; Armingol et al., 2021). Ligand-receptor inference can nominate potential communication pathways, but it does not establish physical interaction, directionality, timing, or causal dependence. Similarly, pseudotime analysis can order transcriptional similarity but should not be interpreted as direct evidence of biological lineage, temporal progression, or protective mechanism without experimental validation.

For these reasons, omics findings in hypoxia-related ischemic tolerance should be treated as prioritization tools. The strongest claims require convergence across spatial localization, protein or metabolic validation, cell-type-specific perturbation, and mechanistic rescue. Recent omics findings are particularly useful for hypothesis generation, but many will require independent replication across platforms, species, ischemia models, time points, and human tissue before being considered mature translational targets.

### 4.5 What multi-omics should do next

The next contribution of multi-omics should be to convert descriptive atlases into validation-ready maps. scRNA-seq, snRNA-seq, and spatial transcriptomics have already identified candidate states and niches (Haque et al., 2017; Lee et al., 2022; Nguyen et al., 2018; Zhang Y. et al., 2024; Liu et al., 2025; Deng et al., 2025; Zhang L. et al., 2024; Lv et al., 2025; Zhang et al., 2026; Han et al., 2024; Garcia-Bonilla et al., 2024; Bormann et al., 2024; Zhao et al., 2026). The next step is same-model, same-time-series integration with proteomics, metabolomics, chromatin accessibility, vascular imaging, and cell type-specific perturbation (Lee et al., 2022; Nguyen et al., 2018; Zhang Y. et al., 2024; Liu et al., 2025; Deng et al., 2025; Zhang L. et al., 2024; Han et al., 2024). That design can separate states that merely track injury from states that actually shift the threshold for tissue survival.

For translational stroke readers, the most important implication is practical: multi-omics should be judged not by the number of cell states it discovers, but by how efficiently it helps prioritize experiments in aged and comorbid stroke models, localize human-relevant targets, identify contradictory or maladaptive programs, and inform stratified intervention design.

## 5 Translational priorities and near-term opportunities

### 5.1 The field is better organized around mechanistic clusters than a single master pathway

The current literature does not support a single upstream switch for hypoxia-related cerebral protection. A better translational strategy is to organize the field around mechanistic clusters and rank them by maturity (Table 3). The most mature cluster links endothelial and BBB stabilization to effective reperfusion and no-reflow biology (Li et al., 2025; Iadecola, 2017; Sweeney et al., 2019; An and Xue, 2009; Wacker et al., 2012; Loan et al., 2024; Roth et al., 2025; Sun et al., 2024). A second cluster centers on astrocyte-mediated metabolic support, especially antioxidant control and lactate shuttling (Guo et al., 2019; Wu et al., 2021; Fadoul et al., 2024; Coimbra-Costa et al., 2021; Hirayama et al., 2024). A third cluster concerns inflammatory threshold setting through microglial and regulated cell-death pathways (Zhan et al., 2021; Lu et al., 2024; Qiu et al., 2024; Ransohoff, 2016; Masuda et al., 2020; Ma et al., 2023; McDonough et al., 2020). HIF/PHD/EPO-centered adaptive programs cut across several of these clusters and remain important candidate upstream regulators (Zhao et al., 2020; Mitroshina et al., 2021; Ruscher et al., 2002; Dong et al., 2022; Madai et al., 2024; Lanigan and O'Connor, 2019; Amin et al., 2021; Zhang P. et al., 2024; Matheoudakis and O'Connor, 2025). Oligodendrocyte-lineage and pericyte programs remain promising but comparatively earlier-stage translational targets (Loan et al., 2024; Roth et al., 2025; Sun et al., 2024; Xu et al., 2019; Suryana and Jones, 2014; Kuwata et al., 2025).

**Table 3:** Prioritized mechanistic clusters for translational stroke research.

Mechanistic cluster	Main cell types	Current evidence strength	Why it matters to stroke translation	Immediate next step
BBB stabilization, endothelial quiescence, and microvascular persistence (Han et al., 2024; Li et al., 2025; Iadecola, 2017; Sweeney et al., 2019; An and Xue, 2009; Wacker et al., 2012; Loan et al., 2024; Roth et al., 2025; Sun et al., 2024)	Endothelial cells; pericytes	High for endothelium; moderate for pericytes	Links conditioning biology to effective reperfusion, hemorrhagic transformation risk, leukocyte trafficking, and no-reflow.	Map protective endothelial/pericyte states across reperfusion windows and test them in aged/comorbid models.
Metabolic support and glutamate control (Zhang et al., 2026; Ma et al., 2022; Escartin et al., 2021; Guo et al., 2019; Wu et al., 2021; Fadoul et al., 2024; Coimbra-Costa et al., 2021; Hirayama et al., 2024)	Astrocytes; neurons	High	Directly influences penumbral energetic failure, excitotoxicity, and region-specific vulnerability.	Define protective astrocyte states in peri-infarct and hippocampal niches and test perturbation-rescue loops.
Inflammatory threshold setting and regulated cell death (Zhan et al., 2021; Lu et al., 2024; Qiu et al., 2024; Ransohoff, 2016; Masuda et al., 2020; Ma et al., 2023; McDonough et al., 2020)	Microglia; macrophages; neurons	Moderate-to-high	Determines whether injury remains containable or becomes self-amplifying.	Anchor state-specific inflammatory programs to tissue outcome and timing rather than broad activation labels.
HIF/PHD/EPO-centered adaptive signaling (Zhao et al., 2020; Mitroshina et al., 2021; Ruscher et al., 2002; Dong et al., 2022; Madai et al., 2024; Lanigan and O'Connor, 2019; Amin et al., 2021; Zhang P. et al., 2024; Matheoudakis and O'Connor, 2025)	Multicellular	Moderate-to-high	Provides a pharmacologically tractable upstream framework intersecting with metabolism, vascular remodeling, and inflammation.	Define dose, timing, hematologic consequences, and cell-type specificity; align biomarkers with protective windows.
White-matter resilience and network preservation (Lv et al., 2025; Zhao et al., 2026; Xu et al., 2019; Suryana and Jones, 2014; Kuwata et al., 2025; Roth and Núñez, 2016)	OPCs; oligodendrocyte-lineage cells	Moderate	Addresses disability depending on network integrity rather than infarct size alone.	Validate adult stroke relevance and combine white-matter endpoints with spatial localization.

This ranking matters because translational research benefits from prioritization. Not every plausible mechanism deserves equal near-term investment. The strongest current rationale lies where mechanistic plausibility, stroke relevance, safety, and feasible endpoints intersect: BBB stability, microvascular patency, metabolic support in penumbral tissue, inflammatory containment, white-matter preservation, and biomarker-defined entry into a protective window.

### 5.2 Clinical translation: constraints from prior neuroprotection failures and acute stroke workflows

The translational path from hypoxia-responsive signaling to stroke therapy is unlikely to be linear. Stroke neuroprotection has a long history of promising preclinical mechanisms failing to produce robust clinical benefit, despite extensive experimental testing and repeated attempts to improve preclinical standards (O'Collins et al., 2006; Stroke Therapy Academic Industry Roundtable (STAIR), 1999; Fisher et al., 2009; Chamorro et al., 2016). This gap reflects multiple factors, including species differences, young and otherwise healthy animal models, insufficient modeling of hypertension, diabetes, atherosclerosis, aging, and small-vessel disease, as well as heterogeneity in stroke subtype, collateral status, infarct location, onset-to-treatment time, and reperfusion status.

Conditioning-based approaches face additional challenges. A protective window must be defined relative to stroke onset, thrombolysis, thrombectomy, reperfusion injury, BBB disruption, inflammatory recruitment, and recovery. Interventions such as intermittent hypoxic conditioning or remote ischemic conditioning must also be feasible within acute stroke workflows, safe in older patients with vascular comorbidities, and compatible with standard reperfusion therapies (Keevil et al., 2024; Li et al., 2024; Chen et al., 2022; Wang et al., 2024). Pharmacological activation of hypoxia-responsive pathways may raise additional concerns related to dose, timing, hematologic effects, vascular permeability, thrombogenicity, and off-target responses (Madai et al., 2024; Lanigan and O'Connor, 2019; Amin et al., 2021; Zhang P. et al., 2024; Matheoudakis and O'Connor, 2025).

Future translation should therefore proceed through an iterative pipeline rather than a direct mechanism-to-treatment model. Candidate mechanisms should first be validated in aged and comorbid models, localized in human stroke-relevant tissue, linked to measurable biomarkers, tested for safety and feasibility, and then evaluated using mechanism-aligned endpoints such as BBB injury, no-reflow, hemorrhagic transformation, effective reperfusion, inflammatory containment, or white-matter preservation.

### 5.3 Intervention strategies should be stratified by paradigm, stage, and population

Potential interventions currently fall into three broad groups. The first includes pharmacological amplification of hypoxia-responsive signaling, including PHD inhibition and related HIF-centered strategies (Madai et al., 2024; Lanigan and O'Connor, 2019; Amin et al., 2021; Zhang P. et al., 2024; Matheoudakis and O'Connor, 2025). The second includes conditioning paradigms themselves, such as IH, IHHE, and RIC/RIPC, which already have supportive preclinical and early clinical literatures in stroke and hypoxic injury (Baillieul et al., 2017; Yuan et al., 2022; Keevil et al., 2024; Li et al., 2024; Chen et al., 2022; Wang et al., 2024). The third includes cell-based or cell-derived approaches, such as hypoxia-preconditioned stromal cells and extracellular vesicles, which may package adaptive signaling into a therapeutically deployable format (De Palma et al., 2025; Wei et al., 2012; Abuzan et al., 2025).

No one of these strategies should be treated as universally applicable. The expected balance of benefit and risk is unlikely to be the same in lifelong high-altitude residents, recently acclimatized lowlanders, older patients with hypertension or diabetes, patients with erythrocytosis, patients receiving thrombolysis or thrombectomy, or individuals in the subacute recovery phase after ischemic stroke. Stroke subtype also matters. Translation therefore requires stratification, not generalization. A conditioning-like intervention should only be proposed after specifying exposure history, disease phase, vascular comorbidity, reperfusion status, hematologic status, safety constraints, and the stroke endpoint it is intended to improve.

### 5.4 A near-term agenda for translational stroke research

#### 5.4.1 Use aged and comorbid models as a default, not an exception

A central limitation of the field is that much of the strongest conditioning evidence still comes from young and otherwise healthy animals, whereas the typical stroke patient presents with aging, hypertension, diabetes, atherosclerosis, and small-vessel disease (Baillieul et al., 2017; Zhu et al., 2025; O'Collins et al., 2006; Stroke Therapy Academic Industry Roundtable (STAIR), 1999; Fisher et al., 2009; Chamorro et al., 2016). This mismatch is no longer a peripheral caveat; it is a major reason why mechanistic coherence may fail to translate. Future validation pipelines should therefore treat age and comorbidity as design variables from the outset.

#### 5.4.2 Move earlier toward human tissue and spatial localization

Human tissue remains the main bottleneck. Stroke-relevant snRNA-seq, spatial transcriptomics, and multimodal profiling now make it feasible to ask whether candidate tolerance-compatible states actually localize to human peri-infarct, white-matter, or hippocampal territories (Liu et al., 2025; Han et al., 2024; Garcia-Bonilla et al., 2024; Bormann et al., 2024; Zhao et al., 2026). This step is critical if altitude-associated biology is to move from conceptual interest to therapeutic relevance.

#### 5.4.3 Build biomarkers for the conditioning window

The field still lacks operational biomarkers that distinguish entry into a protective window from drift toward an injury threshold. EPO, neurofilament light, and related readouts are promising but not yet sufficient as a real-time monitoring framework (Berek et al., 2025). A translationally useful biomarker strategy will likely need to combine circulating markers with vascular, imaging, or perfusion readouts that better reflect BBB stability, microvascular patency, inflammatory state, and hemorrhagic transformation risk.

#### 5.4.4 Design stroke trials around mechanism and context

Early clinical translation should follow mechanism and context rather than the generic proposition that ‘conditioning may help stroke’. The most informative trial designs are likely to be those that predefine disease phase, reperfusion status, vascular comorbidity, biomarker strata, and safety boundaries, and that use endpoints aligned with the mechanism being targeted, such as BBB injury, no-reflow, hemorrhagic transformation risk, effective reperfusion, or white-matter preservation (Keevil et al., 2024; Li et al., 2024; Chen et al., 2022; Wang et al., 2024; Li et al., 2025; Iadecola, 2017; Sweeney et al., 2019; Loan et al., 2024; Roth et al., 2025; Sun et al., 2024).

### 5.5 Scope and limitations of the framework

This review is intentionally conceptual and evidence-graded rather than comprehensive in the systematic-review sense. Its purpose is to organize hypoxia-related stroke hypotheses, not to quantify the overall effect of altitude exposure, conditioning, or individual molecular pathways on clinical stroke outcomes. The proposed evidence tiers and cell-type scores should therefore be interpreted as tools for transparency and prioritization, not as definitive rankings of therapeutic efficacy.

Several limitations follow. First, many mechanistic pathways emphasized here have stronger preclinical than human validation. Second, recent omics studies are valuable but may not yet be broadly replicated across platforms, laboratories, species, ischemia models, and post-ischemic time points. Third, negative, neutral, or maladaptive hypoxic effects may be underdetected when studies are designed around protective hypotheses. Fourth, altitude populations differ in ancestry, exposure duration, socioeconomic context, altitude level, comorbidity, and access to acute care. These factors make it inappropriate to infer generalized stroke protection from altitude biology alone.

**Figure 3:**
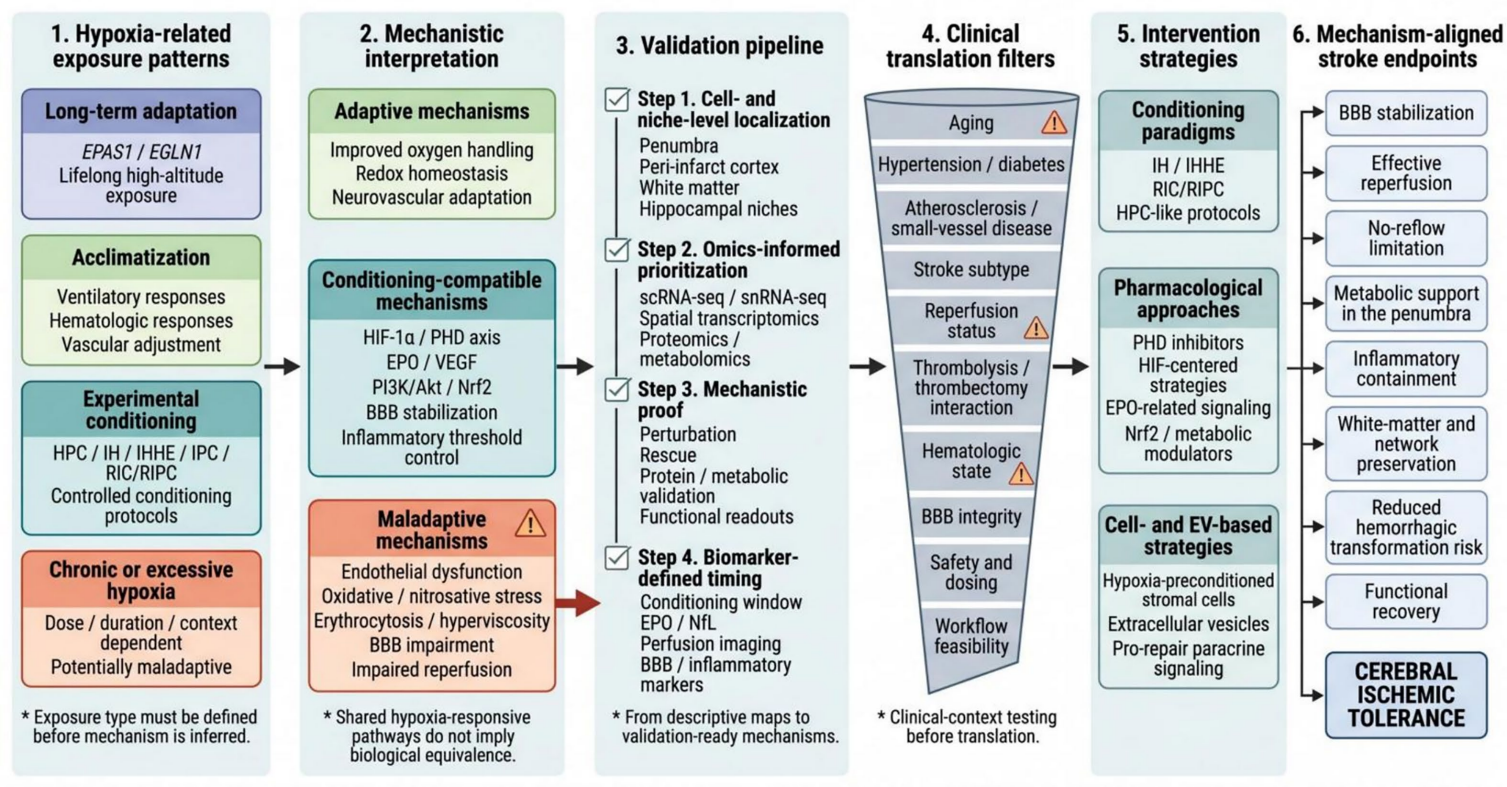
Integrative translational schematic. Hypoxia-related exposure patterns should first be defined and separated into adaptive, maladaptive, and conditioning-compatible mechanisms. Candidate programs then require cell- and niche-level validation, mechanistic proof, biomarker-defined timing, and testing under clinical filters such as aging, comorbidity, reperfusion status, thrombolysis/thrombectomy interaction, hematologic state, BBB integrity, workflow feasibility, and mechanism-aligned endpoints.

The strongest use of the framework is therefore hypothesis refinement. It identifies where evidence is direct, where it is supportive, where it is only indirect, and where explicit falsification is needed. The framework should be updated as replicated human tissue data, mechanistic rescue experiments, and clinically feasible conditioning or pharmacological trials become available (Figure 3).

## 6 Conclusion

High-altitude-associated hypoxic cues are informative for cerebral ischemic tolerance research and may engage parts of its biology, but they do not yet prove that a naturally occurring altitude-induced tolerant state exists in humans. Experimental HPC remains the clearest direct evidence that a defined sublethal hypoxic stimulus can induce a time-limited tolerant state (Zhu et al., 2025; Darlington et al., 2021; Zhao et al., 2020). Human altitude epidemiology, physiology, and genetics are essential for boundary setting and pathway nomination, but they remain indirect unless linked to stroke-specific endpoints.

For translational stroke research, the value of altitude-related biology lies less in labeling altitude as protective and more in organizing a sharper hypothesis space. The strongest current priorities are endothelial and BBB stabilization, effective reperfusion and no-reflow biology, astrocyte-mediated metabolic support, inflammatory threshold control, and white-matter resilience within defined tissue niches. Multi-omics is central to that agenda when it is used to prioritize perturbation-ready targets rather than to inflate descriptive certainty (Haque et al., 2017; Lee et al., 2022; Nguyen et al., 2018; Zhang Y. et al., 2024; Liu et al., 2025; Deng et al., 2025; Zhang L. et al., 2024; Lv et al., 2025; Zhang et al., 2026; Han et al., 2024; Garcia-Bonilla et al., 2024; Bormann et al., 2024; Zhao et al., 2026; Li et al., 2025; Iadecola, 2017; Sweeney et al., 2019; An and Xue, 2009; Wacker et al., 2012; Loan et al., 2024; Roth et al., 2025; Sun et al., 2024).

The next step for the field is explicit and testable: separate adaptation from acclimatization, chronic hypoxia, and conditioning; grade evidence according to what it can actually support; localize candidate mechanisms in stroke-relevant human tissue; account for maladaptive hypoxic biology; and evaluate interventions in the populations and disease phases in which they are biologically and clinically plausible. That agenda is more likely to advance stroke translation than any broad claim that hypoxia, by itself, protects the brain.
